# Igniting the Fire: *Staphylococcus aureus* Virulence Factors in the Pathogenesis of Sepsis

**DOI:** 10.1371/journal.ppat.1003871

**Published:** 2014-02-13

**Authors:** Michael E. Powers, Juliane Bubeck Wardenburg

**Affiliations:** 1 Department of Microbiology, The University of Chicago, Chicago, Illinois, United States of America; 2 Department of Pediatrics, The University of Chicago, Chicago, Illinois, United States of America; Duke University Medical Center, United States of America

Sepsis is a devastating disease process characterized by a systemic inflammatory response in the host, evoked by a known or suspected pathogen. *Staphylococcus aureus* has emerged as a leading etiologic agent of sepsis, owing to its propensity to cause deep-seated tissue infection and bacteremia [1]. *S. aureus* harbors an arsenal of virulence factors to facilitate tissue adhesion, immune evasion, and host cell injury. In the bloodstream, these factors cause inflammation, impair immune cell function, alter coagulation, and compromise vascular integrity. This review will discuss key secreted and surface-anchored proteins required for *S. aureus* infection in the hostile host environment of the bloodstream, emphasizing mechanistic insights on virulence factor function that illustrate the complex nature of the host–pathogen interaction. While we currently lack a clear understanding of the temporal and spatial integration of these virulence factors in the bloodstream, it is apparent that *S. aureus* triggers pathophysiologic disturbances that are further amplified by the host inflammatory response, culminating in the severe clinical manifestations of sepsis and septic shock.

## Inflammation: An Early *S. aureus* Insult in Sepsis

The clinical manifestations of sepsis span a continuum of severity, in the most extreme form termed “septic shock,” in which vascular insults and systemic inflammation lead to compromised cardiac function and blood pressure, culminating in impaired oxygen delivery to the tissues and organ failure. In the United States, ∼750,000 individuals suffer from sepsis per year, with mortality rates that approach or even exceed 50% in severe disease [2]. Multiple clinical trials aimed at curbing the host inflammatory response to severe infection have yielded limited clinical success [3]. The mainstay of therapy for sepsis and septic shock remains 2-fold: (1) rapid treatment of the underlying infection and (2) early resuscitation to blunt physiologic abnormalities that potentiate disease progression [4]. The nature of these beneficial interventions focuses attention on rigorously defining the inciting insult caused by the pathogen.

**Figure 1:**
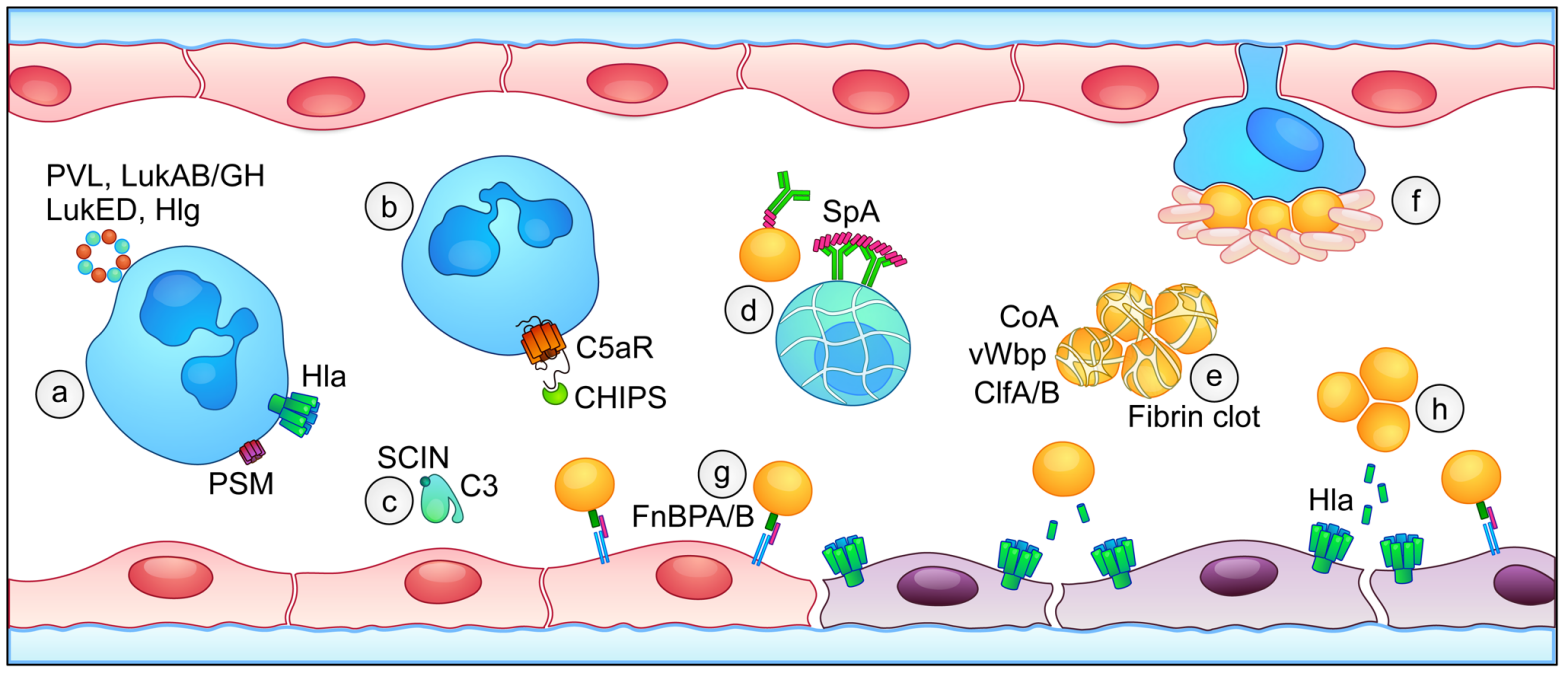
Overview of *S. aureus* virulence factors that contribute to the pathogenesis of sepsis. (a) Leukocytes are targeted and injured by bi-component leukocidins (PVL, LukAB/GH, LukED, and Hlg, blue and orange), phenol-soluble modulins (PSM, purple), and α-toxin (Hla, green). (b) Inhibition of host complement pathways occurs through Chemotaxis Inhibitory Protein of Staphylococci (CHIPS) binding to the C5a receptor and (c) Staphylococcal Complement Inhibitor (SCIN)-mediated blockade of C3 convertase activity. (d) Staphylococcal protein A (SpA) binds to host antibodies, preventing opsonophagocytosis and contributing to apoptotic death of B cells. (e) Coagulase (Coa) and von Willebrand factor binding protein (vWbp) initiate fibrin clot formation, facilitating the formation of staphylococcal aggregates in the blood through the action of clumping factors A and B (ClfA/B). (f) Platelet traps surround staphylococci that adhere to macrophage-like Kupffer cells in the liver sinusoid. (g) Fibronectin-binding proteins A and B (FnBPA/B) bind to integrin α5β1, enabling the tethering of *S. aureus* to endothelial cells in the context of blood flow. (h) Expression of *S. aureus* α-toxin (Hla) causes direct injury to the endothelium, disrupting the integrity of the endothelial barrier.

Multiple *S. aureus* proteins and cell wall components are pro-inflammatory, eliciting host responses similar to gram-negative lipopolysaccharide (LPS) [5]. The production of cytokines, including TNF-α and IL-6, results from the action of *S. aureus* lipoproteins on mononuclear phagocytes through TLR-2 pathway activation [6], [7]. Furthermore, bloodstream exposure of rat hosts to peptidoglycan and lipoteichoic acid leads to induction of IL-1 and IFN-γ [8]. Toxin-induced cellular injury also elicits prominent host inflammatory responses. In the bloodstream, circulating immune cells and the vascular endothelium are primary targets of staphylococcal virulence factors. Among the longest studied of these toxins are the staphylococcal superantigens that potently stimulate non-specific T-cell proliferation and activation and potentiate the host inflammatory response associated with sepsis [9]. A family of bi-component leukotoxins including Panton-Valentine Leukocidin (PVL), Leukocidin AB/GH (LukAB/GH), Leukocidin ED (LukED), and γ-hemolysin (Hlg) injure an array of leukocytes including neutrophils, mononuclear phagocytes, and T cells [10]. Also contributing to leukocyte injury is the family of cytolytic peptides termed phenol soluble modulins (PSMs) [11] and the small pore-forming toxin α-hemolysin (α-toxin, Hla) (Figure 1a) [12]. Genetic regulatory control that leads to increased production of PVL, PSMs, and α-toxin in highly virulent methicillin-resistant *S. aureus* (MRSA) strains has been described as a molecular mechanism that underlies increased disease severity observed upon infection with these isolates [13]–[15]. These virulence factors are potent stimulants of leukocyte inflammatory responses [10], [12], [16]. *S. aureus* mutants engineered to lack expression of even one of these toxins exhibit virulence defects in experimental infection [11], [17]–[19], suggesting that the collective impact of this group of toxins on inflammation and destabilization of the host during bloodstream infection is substantial.

## Escape from Innate Immune Cells: A Key to Bloodstream Survival

In concert with direct leukocyte injury, *S. aureus* utilizes a number of strategies to modulate the innate host immune response and prevent bacterial clearance in the bloodstream [20]. Most clinical *S. aureus* isolates express a polysaccharide capsule that affords protection against phagocyte-mediated clearance; furthermore, the pathogen is able to resist killing in the phagocyte. *S. aureus* also secretes factors that function to dampen leukocyte recruitment or prevent opsonophagocytic uptake as a predecessor to intracellular killing. Two of the best-studied of these proteins are CHemotaxis Inhibitory Protein of Staphylococci (CHIPS) and Staphylococcal Complement INhibitor (SCIN). CHIPS binds to the cellular receptors for C5a and N-formyl peptides, diminishing the ability of both bacterial peptides and complement activation to function as leukocyte chemoattractants (Figure 1b) [21], [22]. SCIN potently inhibits human complement pathway defenses by blocking C3 convertase activity, reducing the deposition of the C3b opsonin on *S. aureus* and decreasing neutrophil uptake and killing [23] (Figure 1c). Both of these staphylococcal virulence factors display human specificity and are secreted during early bacterial growth to provide rapid evasion of innate immunity [24].

To limit the function of innate immune cells that successfully circumvent anti-chemotactic signals, *S. aureus* utilizes the cell-wall–anchored Staphylococcal protein A (SpA) to preclude antibody-driven opsonophagocytic clearance. SpA binds to the F_c_ and F_ab_ regions of host antibodies [25], preventing staphylococcal antigen recognition and F_c_-mediated effector functions. SpA also engages the B cell receptor and initiates activation-induced apoptotic death of V_H_3^+^ B cells (Figure 1d) [26]. *S. aureus* SpA mutants display virulence defects in an intraperitoneal model of lethal infection as well as intravenous infection that leads to arthritis and renal abscess formation [27]–[29]. Immunization with an inactive, “non-toxinogenic” SpA variant is protective in lethal *S. aureus* sepsis, promoting opsonophagocytic clearance in the blood [28]. In addition to these immunoevasion strategies and the broadly toxic effects of the array of staphylococcal toxins on host immune cells [10]–[12], recent studies indicate a prominent role for LukED in bloodstream infection by virtue of its ability to injure monocytes and lymphocytes, diminishing phagocytic uptake and promoting pathogen dissemination [18], [30]. Collectively, these bacterial defense mechanisms increase the burden of staphylococci in the blood, further compromising the septic host.

## Modulation of Intravascular Coagulation: A Host–Pathogen Tug-of-War

Coagulopathy is another hallmark of septic shock, manifest as pathologic clotting within the microvasculature and a predisposition toward systemic bleeding [3]. *S. aureus* encodes virulence factors that modulate both soluble and cell-mediated pathways of coagulation. Physiologic coagulation in response to injury requires the rapid, localized activation of platelets, together with activation of the soluble clotting cascade. This serine protease-based cascade culminates in the enzymatic conversion of prothrombin to thrombin, in turn promoting the cleavage of fibrinogen to soluble fibrin monomers that polymerize into insoluble fibrin. Platelet-fibrin matrices form a physical substrate for plugging of the injured vasculature. While these processes are tightly regulated to ensure hemostasis yet avoid untoward intravascular thrombosis, bacterial virulence factors and the underlying host inflammatory state in sepsis induce pathologic alterations of coagulation. The staphylococcal virulence factors coagulase (Coa) and von Willebrand factor binding protein (vWbp) promote the non-catalytic activation of prothrombin, yielding cleavage of soluble fibrinogen to engender fibrin clot formation in the absence of an inciting injury [31], [32]. Fibrin clots promote clumping factor protein-mediated (ClfA and ClfB) aggregation of staphylococci (Figure 1e) [27]. Agglutination of *S. aureus* in the blood promotes bacterial survival, noted by the significant virulence defect in *S. aureus* strains that lack expression of Coa, vWbp, and ClfA [33]. In this context, survival is favored by protection of the aggregated organisms against phagocytic clearance. As activation of host coagulation pathways is proinflammatory [34], manipulation of this pathway by *S. aureus* likely contributes to the systemic inflammatory response.

Recent observations indicate the importance of thrombosis in immunodefense [35], demonstrating that platelets confer anti-staphylococcal immunity to bloodstream infection [36]. Intravital imaging revealed that platelets form aggregates around staphylococci adhered to macrophage-like Kupffer cells associated with the liver sinusoidal endothelium, entrapping *S. aureus* and facilitating pathogen clearance (Figure 1f) [36]. Suggesting the host-protective role of these traps, experimental platelet depletion leads to increased mortality from bloodstream infection [36]. While the role of platelets as innate immune cells has recently gained considerable attention, the modulation of normal platelet function by *S. aureus* has long been suggested by the ability of α-toxin to initiate platelet activation and aggregation [37]. α-toxin–induced platelet activation would thereby seem to promote the formation of platelet traps and support bacterial clearance—a paradoxical “anti-virulence” effect, highlighting the need to further investigate the precise molecular mechanisms by which staphylococcal virulence factors modulate platelet function in innate immunity.

## The Microvascular Endothelium: A Site of Coordination?

Blood flow presents a major hurdle to both the pathogen and the host during intravascular infection. The ability of immune cells to identify and then contain *S. aureus* in the context of a branching vascular tree and the dilutional effects of blood flow is challenged. Conversely, the pathogen is challenged to (1) constrain the delivery of soluble virulence factors that initiate pathologic coagulation in flowing blood and (2) overcome fluid shear stress to adhere to the vascular wall and promote dissemination. Fibronectin-binding protein A (FnBPA) is a surface-displayed protein that facilitates endothelial adherence (Figure 1g) [38]. Variations in FnBPA underlie differences in fibronectin-binding affinity; high-affinity variants enhance endothelial cell binding and correlate with increased endothelial invasion in bloodstream infection [38]. The initial tethering of *S. aureus* to the endothelium may favor the establishment of a microenvironment in the small vessels or slow-flow vessels, such as liver sinusoids, in which *S. aureus*–induced coagulation and aggregate formation enhances the localized elaboration of virulence factors (Figure 1h) [39].

A primary molecular mechanism for bacterial-induced vascular permeability is endothelial disruption due to extreme inflammation [34]. In addition to its role in leukocyte and platelet injury, recent studies suggest that α-toxin co-opts the function of its cellular receptor A Disintegrin and Metalloprotease 10 (ADAM10) to disrupt the endothelial barrier by promoting the untimely cleavage of vascular endothelial (VE)-cadherin, destroying the inter-cellular junction that is required for vascular integrity [17]. As an intact endothelium forms the principal physical barrier to intravascular dissemination of bacterial pathogens, disruption of this barrier is expected to promote dissemination, a common and severe consequence of *S. aureus* sepsis. Damaged endothelium is also a potent stimulus for the rapid recruitment of platelets and activation of soluble clotting cascades. The microvascular endothelial surface may, thus, form a site wherein the pathogen, its armamentarium of virulence factors, platelets, leukocytes, and host coagulation proteins are co-localized. This “coordination” site may simultaneously trigger microvascular occlusion and increased vascular permeability—events that decrease effective blood flow to the tissues and precipitate sepsis-associated vital organ failure.

## Towards the Future: Insights That May Change *S. aureus* Sepsis

The devastating mortality of sepsis and the inability of current clinical approaches to mitigate disease testify to our limited understanding of the complex host–pathogen interaction in the bloodstream. While the virulence factors discussed herein each contributes to sepsis pathogenesis, loss of any one factor is insufficient for complete protection against experimental *S. aureus* challenge. Similarly, vaccine approaches that target isolated virulence factors do not provide complete protection against lethal sepsis [40]. Together, these observations highlight our need to understand the temporospatial regulation of virulence factor expression and action in vivo. While exaggerated host inflammatory responses are associated with the progression of severe septic shock, insults directly delivered by *S. aureus*—coagulopathy, immune cell injury, microvascular occlusion, and barrier damage—collectively mirror disease endpoints in sepsis. These insults likely initiate the very pathophysiologic state that is then exacerbated by an untoward host response. While multiple blood-borne bacteria incite endothelial injury and lead to a coagulopathic state manifested as sepsis [41], whether a deliberate pathogen-driven coordination of these events occurs to promote virulence requires further study. The host–pathogen interaction in the bloodstream has proven extremely challenging to redirect in favor of the host, in spite of the commonality of these observed physiologic disturbances. The essential role of bacterial virulence factors as catalysts of sepsis, however, suggests that a keen focus on understanding how these factors are integrated in time and space within the vasculature should yield new insights for sepsis prevention and therapy in the coming years.
